# Genetic and Morphological Variation of the Forkbeard, *Phycis phycis* (Pisces, Phycidae): Evidence of Panmixia and Recent Population Expansion along Its Distribution Area

**DOI:** 10.1371/journal.pone.0167045

**Published:** 2016-12-12

**Authors:** Ana Rita Vieira, Ana Sofia B. Rodrigues, Vera Sequeira, Ana Neves, Rafaela Barros Paiva, Octávio S. Paulo, Leonel Serrano Gordo

**Affiliations:** 1 MARE - Marine and Environmental Sciences Centre, Faculdade de Ciências, Universidade de Lisboa, Lisboa, Portugal; 2 Departamento de Biologia Animal, Faculdade de Ciências, Universidade de Lisboa, Lisboa, Portugal; 3 cE3c - Centre for Ecology, Evolution and Environmental Changes, Faculdade de Ciências, Universidade de Lisboa, Lisboa, Portugal; University of Innsbruck, AUSTRIA

## Abstract

The knowledge of population structure of a species is essential to effectively assess and manage fisheries. In the present study, genetics, by mitochondrial DNA cytochrome *b* sequence analysis, and body geometric morphometrics were used to evaluate the existence of distinct populations of the forkbeard (*Phycis phycis*), an important commercial species in several European countries, especially Portugal and Spain. For geometric morphometric analysis, specimens were collected in the Northeast Atlantic Ocean—Azores, Madeira and mainland Portugal, and for genetic analysis, these samples were complemented with samples collected in the Mediterranean Sea—Spain, Italy and Croatia, in order to cover the entire distribution area of the species. Body shape of the forkbeard from the Northeast Atlantic was found to be highly variable. This variation was probably associated with different environmental factors between the study areas. Despite morphological variation, a low genetic differentiation between samples from different areas was found, most likely due to gene flow that occurred in the past or with the demographic history of the species. Moreover, the presence of unique haplotypes in the Northeast Atlantic and in the Mediterranean suggests that recent gene flow between populations from these areas should be limited. Altogether, a high haplotype diversity, a low nucleotide diversity, a “star-like” network and the results of the mismatch distribution, indicate a possible signature of recent population expansions, which probably started during the end of the Last Glacial Maximum and led to the colonization of the Northeast Atlantic and the Mediterranean.

## Introduction

In marine environments, population genetic structure is influenced by life-history traits and species-specific ecological supplies and, therefore, genetic differentiation in marine organisms is highly influenced by their dispersal capability [1]. The theory of population genetics has suggested that marine organisms, such as fishes, that have a very short larval phase or a direct development, most likely have low dispersal ability, with consequent restrict genetic exchange between populations [2]. In contrast, species with a long larval stage probably have high dispersal ability, promoting interchanges of individuals and genes between different geographical regions [3], resulting in genetic homogeneity in populations [4]. Several factors, such as larval behavioural patterns, marine currents, coastal topography, temperature regimes and ecological requirements, may have a strong effect in the genetic structuring of marine organisms [3, 5, 6], even though the marine environment provides passive dispersal opportunities [7]. In the marine environment, relationships between populations are presumably the consequence of complex and dynamic interactions between the biological and physical environments, and the life histories, behaviour and physiology of individual taxa [2].

Morphological variation of populations can be caused by additive genetic variation or processes of phenotypic plasticity in response to different selective pressures or environmental constraints [2, 8]. For fishes, in particular, body shape has been interpreted as a result of interaction between genetic and environmental factors [9, 10], especially during early development stages. Two different sources of morphometric variance are produced by ontogeny: isometric size variation due to growth and allometric shape variance due to developmental change in form [11].

A recommended procedure for population identification is the use of a holistic approach where, at least, one phenotypic-based method and one genetic technique should be used [12]. The combination of such methods is crucial to obtain a more complete and precise view on the basis of population differentiation and structure, which are essential to effectively assess and manage natural resources [13]. A failure to recognize the population structure of an exploited species can lead to erroneous management actions, including the overexploitation and depletion of less productive stocks [14].

In the present study, a comparison between variation and adaptation at the morphological and genetic levels was performed, using a fish species: the forkbeard, *Phycis phycis* (Linnaeus, 1766). This species is a gadiform benthopelagic fish with a wide distribution in the Northeast Atlantic (from the Bay of Biscay to Morocco and Macaronesia islands) and in the Mediterranean Sea [15]. The forkbeard inhabits on rocky bottoms and sandy-muddy bottoms near rocks at depths up to 650 m [15], taking refuge in holes during the day and becoming an active predator at night [15–17]. In the southern NE Atlantic, the forkbeard is an important commercial species in both Portugal and Spain [18–20], with the landings reaching about 800 tons per year in Portugal [21] and about 4,000 tons per year in Spain [22]. In the Mediterranean, data is only available for Italy, with landings reaching about 300 tons per year [22]. The forkbeard is one of the most important gadiform species commercially exploited by the Portuguese fleet although its fishery is still classified as developing fishery [18–20]. Despite its economic importance, the population structure of forkbeard is still unknown. The only published study on this subject, based on otolith shape analysis, showed the possible existence of more than one stock unit of the forkbeard in the Northeast Atlantic, suggested by the separation of populations from Azores, Madeira and mainland Portugal [19].

The aim of this study was to investigate the population structure of the forkbeard along its distribution area, based on genetic diversity and differentiation, as well as body shape variation. A phylogeographical and population genetic study was performed based on the analysis of fragments of mitochondrial DNA (mtDNA) cytochrome *b* gene (cyt *b*) and of the first intron of the nuclear S7 ribosomal protein gene (S7). These genes have been widely used in both genetic structure of fish populations (e.g. [23–25]) and fish phylogeographical and demographic studies (e.g. [25–28]), proving to be useful in revealing historical and present-day barriers to gene flow in widespread fish species [29]. Variation in body shape of the forkbeard was analysed using geometric morphometrics (by landmark analysis), another tool commonly used in fish population differentiation (e.g [30–36]). Through both genetic and morphological data, patterns of variation between populations could be interpreted.

## Materials and Methods

### Ethics statement

Sampling for the present study focused on the forkbeard from the Northeast Atlantic Ocean and the Mediterranean Sea. Ethical or government approval, specific permissions or licenses for sample collection were not required for this study, as all specimens were collected as part of routine fishing procedures by fishermen of commercial fleet. As the forkbeard is a deep-water species, fish are killed during the hauling of fishing gear due to differences in atmospheric pressure. No animals were killed specifically for this study. Dead specimens were acquired in the commercial ports of Horta (Azores, Portugal), Funchal (Madeira, Portugal), Peniche (mainland Portugal), Fuengirola (Spain), Fiumicino (Italy) and Split (Croatia). Sampling was performed in the laboratory. The forkbeard is not an endangered or protected species.

### Sampling

**Fig 1:**
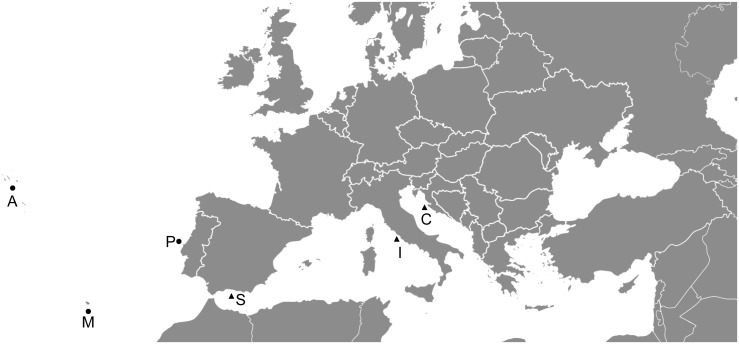
Sampling locations of the forkbeard in the Northeast Atlantic Ocean and the Mediterranean Sea. A—Azores archipelago; M—Madeira archipelago; P—mainland Portugal; S—Spain; I—Italy; C—Croatia. Black dots—samples collected for both geometric morphometric and genetic analyses. Black triangles—samples collected for genetic analysis (adapted from Wikimedia Commons, https://commons.wikimedia.org).

For geometric morphometric analysis, forkbeard specimens were collected from commercial landings of fishing vessels operating off Azores and Madeira archipelagos, and mainland Portugal—Northeast Atlantic (Fig 1), between November 2011 and March 2013. All specimens were deep-frozen (-18°C), stored in a horizontal position to avoid any deformation of the body until the time of the analysis, and thawed before being analysed. Only fish with mouth closed and with no body deformation were analysed. For each fish, total length (TL, to the nearest 0.1 cm) and sex were recorded. A total of 268 adult and sexually mature fish (TL > 40 cm) were analysed: 79 from Azores (41.2–52.6 cm TL; 36 females and 43 males), 88 from Madeira (40.8–58.7 cm TL; 36 females and 49 males) and 101 from mainland Portugal (40.0–52.1 cm TL; 64 females and 37 males).

For genetic analysis, a piece of fin of sampled specimens from each population from the Northeast Atlantic—Azores (n = 29), Madeira (n = 26) and mainland Portugal (n = 27) was collected and preserved in 96% ethanol. In order to cover the entire distribution area of the forkbeard, fins of specimens from 3 populations from the Mediterranean Sea—Spain (n = 17), Italy (n = 2) and Croatia (n = 26) were also collected (Fig 1), preserved in 96% ethanol and shipped to the laboratory.

### Geometric morphometric analysis

#### Data acquisition

**Fig 2:**
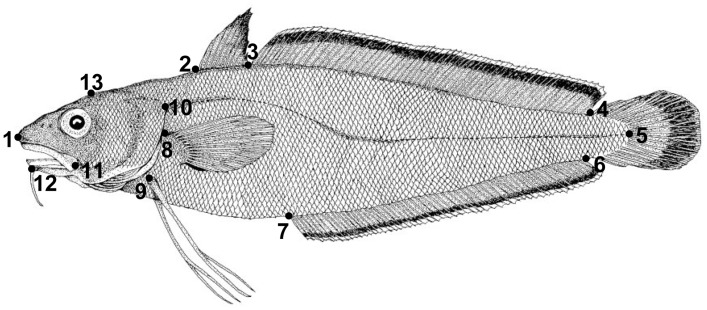
Anatomic landmarks used on forkbeard geometric morphometric analysis. 1 –snout tip; 2 –base of the first dorsal fin; 3 –between first and second dorsal fins; 4 –posterior end of the second dorsal fin; 5 –end of lateral line; 6 –posterior end on anal fin; 7 –anterior insertion of the anal fin; 8 –superior insertion of the pectoral fin; 9 –insertion on the pelvic fin; 10 –posterior limit of the operculum; 11 –mid-point of the posterior extremity of upper-jaw; 12 –insertion of the barbell; 13 –top of the head.

To quantify body shape variation of the forkbeard from Northeast Atlantic, a total of 13 anatomical landmarks were defined (Fig 2), which correspond to hard structures (e.g. fin insertion points) mostly distributed along the fish body contour in order to be meaningful in systematic terms [11]. Each landmark was fixed on the left side of each specimen and images were acquired using a Canon EOS 350D digital camera, with a fixed focus of 50 mm lens to avoid image distortion, coupled to a computer. Then, each anatomical landmark was transformed into Cartesian coordinates using the software tpsDig version 2.16 [37].

#### Geometric morphometrics

All sampled specimens from the Northeast Atlantic were analysed by geometric morphometrics (n = 268) (see [38–42] for more detailed description of the method).

To remove non-shape variation, a generalized Procrustes superimposition was applied to the landmark configurations [43]. This corresponds to fitting a very simple model, taking into account only translation, rigid rotation, scale and possibly uniform shape change for the differences in landmark configurations [43], in order to minimize the sum-of-squared distances (Procrustes distances) between homologous landmarks of all specimens. Given the different length ranges of the samples from the three study areas, a multivariate regression of the Procrustes coordinates on centroid size was performed [44] to remove the size effect and possible allometric relationships between variables. The residuals of this regression were used as “size-free” variables in following statistical analyses.

The existence of sexual dimorphism within the study areas was determined by the quantification of differences between the mean shape of males and females using the Procrustes distance. A permutation test using 10,000 runs was used to assess the null hypothesis of no differences between shape of males and females from the three study areas.

Canonical variate analysis (CVA) was performed to detect morphometric differences in the body shape of forkbeard from the three study areas and to investigate if body shape could be used to classify samples in terms of area of origin. Pairwise comparisons of mean shapes of forkbeard from different areas were based on Procrustes distances, and a permutation test with 10,000 runs was used to assess the null hypothesis of no difference between samples [35].

The mean shapes of the forkbeard from the three study areas were estimated by warped outline drawings, which were performed using the wireframe function [45].

All geometric morphometric analyses were done in the software MorphoJ version 1.05c [45]. A significance level of 0.05 was set for all the statistical analysis.

#### Statistical analysis

Canonical discriminant analysis (CDA) was also performed to detect morphometric differences in body shape of the forkbeard from the three study areas. The Jackknife cross-validation was used to calculate an unbiased estimation of classification success, being each specimen allocated to the group with the nearest centroid and the proportion of correctly allocated specimens calculated [46]. The discriminatory effectiveness of the analysis was also calculated using the Wilks’ lambda (λ) [47] and the Cohen’s Kappa (κ) [48] (the latter provides more reliable results than the former [49]). These statistical analyses were performed in IBM SPSS Statistics 20. The significance level was set at 0.05 for all the statistical tests used.

### Genetic analysis

#### DNA extraction, amplification, sequencing and alignment

Genomic DNA was extracted from fins preserved in 96% ethanol using the E.Z.N.A. Tissue DNA Kit (Omega Bio-Tek), according to the manufacturer's instructions. An 899 bp fragment of mitochondrial DNA (mtDNA) cytochrome *b* (cyt *b*) gene was amplified by polymerase chain reaction (PCR) for all 127 forkbeard specimens. A pair of primers purposely designed by the authors for this study, based on 3 aligned cyt *b* complete sequences of *Phycis phycis* obtained from GenBank (http://www.ncbi.nlm.nih.gov/genbank) (accession numbers: EU036476, EU036477 and DQ197978) was used to amplify the region of interest: FORcytbF (forward) 5’-GCCAGCCTTCGAAAAACAC-3’ and FORcytbR (reverse) 5’-GCTTCGTTGTTTTGAGGTGTG-3’. In addition, PCR amplification of a 751 bp fragment of the first intron of the nuclear S7 ribosomal protein gene (S7) was performed with the following pair of primers: S7RPEX1F (forward) 5’-TGGCCTCTTCCTTGGCCGTC-3’ and S7RPEX2R (reverse) 5’-AACTCGTCTGGCTTTTCGCC-3’ [50].

PCR amplifications were performed in a 20 μl total reaction volume with 0.6 μM of each primer, 0.175/0.2 mM dNTPs, 0.925/1.125 mM MgCl_2_, 1.4/1.2 μl BSA (10 ng/μl), 4.0 μl 5x Colorless GoTaq Flexi Buffer, 0.05 U GoTaq DNA Polymerase (Promega) and 2.0 μl of template DNA (values for cyt *b*/S7, respectively). PCR conditions used were composed by an initial denaturation step at 95°C for 5 min, followed by 35 cycles of denaturation at 95°C for 45 sec, annealing at 55°C or 68°C, for cyt *b* or S7, respectively, for 35 sec and extension at 72°C for 1 min, with a final extension period at 72°C for 10 min. Amplification results were confirmed by 1% TBE agarose gel electrophoresis. All PCR products were purified with the SureClean kit (Bioline) following the manufacturer’s protocol, and sequenced in the forward and the reverse directions (using the same primers) by an ABI PRISM 3700 DNA analyser at Macrogen (http://www.macrogen.com). The existence of haplotypes present in one single specimen was confirmed by performing independent PCR amplifications and sequencing (as previously described). The obtained cyt *b* and S7 sequences were compared against a database in GenBank using the basic local alignment search tool (Blast) (http://blast.ncbi.nlm.nih.gov/Blast.cgi) to confirm that they belong to both genes.

Fragment sequences of both genes were verified and edited using the software Sequencher version 4.0.5 (Gene Codes Corporation) and BioEdit version 7.0.9 [51]. For each specimen, the existence of unique nucleobases for each sequence position was confirmed by analysing the chromatograms, excluding any possible occurrence of errors in the base calling and any evidence of pseudogenes. All sequences of both genes were aligned using ClustalX version 2.1 [52] and converted into the appropriate format with Concatenator version 1.0.1 [53].

#### Genetic data analysis

Data analysis of nuclear S7 was suspended because this gene did not show genetic variation.

Two different geographical groups were defined *a priori*: Northeast Atlantic Ocean, composed by samples of Azores, Madeira and mainland Portugal, and Mediterranean Sea, constituted by samples of Spain, Italy and Croatia.

The number of polymorphic sites and haplotypes, haplotype diversity (*h*), and nucleotide diversity (π) were calculated for each sampled population, as well as for both geographic groups and for the total samples, using the software DnaSP version 5.10.01 [54]. Maximum parsimony median-joining haplotype network [55] was created using Network version 5.0.0.0 (Fluxus Technology Ltd.). Analysis of molecular variance (AMOVA), with 10,000 permutations, was performed using Arlequin version 3.5.1.2 [56] to assess population genetic structure. This analysis produces estimates of variance components and F-statistic analogues, reflecting the correlation of haplotypes at different levels of hierarchical subdivision [57].

Mismatch distribution (frequency distribution of the number of pairwise differences among haplotypes) was used to detect and estimate the timing of population expansion [58]. Estimated expansion values were calculated in Arlequin software and graphics of frequency distribution were performed using DnaSP. Populations that have been historically stable are predicted to have multimodal mismatch distributions, whereas those that have undergone a recent expansion are predicted to be unimodal [59]. To test the observed mismatch distribution goodness-of-fit to the expansion model and to obtain confidence intervals around the estimated mode of mismatch distribution, 1,000 permutation replicates were used [60]. Statistically significant differences between observed and expected distributions were evaluated with the sum of the square deviations (SSD) and Harpending’s raggedness index (raggedness) [61, 62]. Neutrality tests of Tajima’s D [63] and Fu’s F-statistics [64] were performed in Arlequin to detect changes in population size and/or estimating deviations from neutrality, assuming a constant population size at mutation-drift equilibrium [2]. Thus, evidence of expanding populations was assumed when significant negative values of Tajima’s D and Fu’s F-statistics were obtained [65, 66].

Time since population expansion in years (*t*), as well as the 95% confidence interval, was calculated from τ = 2*ut*, where τ is the expansion time parameter generated by Arlequin and *u* is the mutation rate per nucleotide per year multiplied by sequence length (i.e. number of nucleotides) [58, 62]. A generation time of one year was assumed, as the forkbeard spawns every year [20], and a mutation rate of 3.86% per million years was considered, based on other gadiform species [67], since no information on mutation rate of the forkbeard is available.

## Results

### Geometric morphometric analysis

In this study, a similar length range of specimens from the Northeast Atlantic was used, based only in adult mature specimens. However, and despite the fact that juveniles and very large specimens were excluded from analyses to minimize the effect of size on shape, a very small amount, but statistically significant (*p* < 0.031) of shape variation due to allometric growth was detected (1.16% of total shape variation). Therefore, data were allometrically corrected to remove the size effect between variables and the residuals were used as “size-free” variables in the statistical analyses.

No statistically significant differences were found between males and females shape within the three study areas from the Northeast Atlantic (*p*_Azores_ = 0.094; *p*_Madeira_ = 0.075; *p*_Mainland_ = 0.268), so sexes were analysed together for each geographical area.

**Fig 3:**
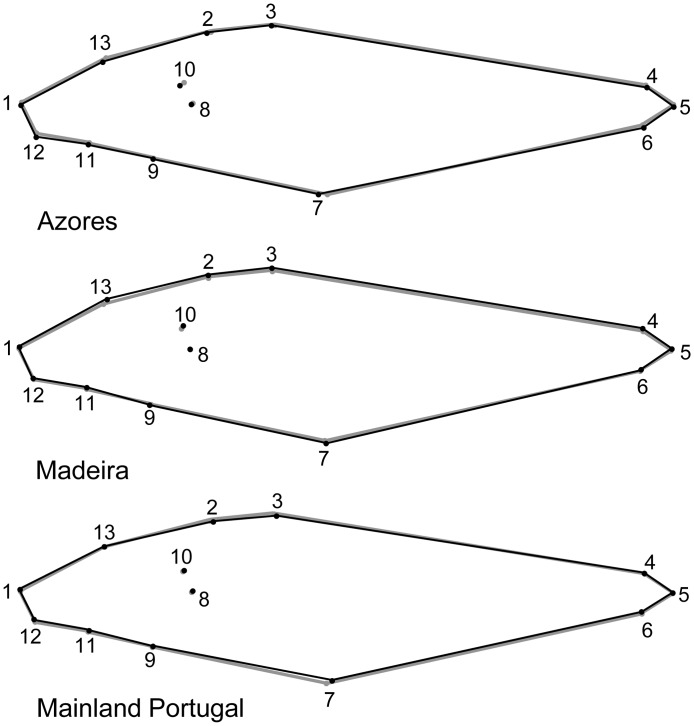
Mean body shapes of the forkbeard from the three study areas from the Northeast Atlantic. Each image represents the transformation from the overall mean shape (grey line) to the mean shape for each location (black line). Numbers represent anatomic landmarks identified in Fig 2.

Statistically significant differences were found in the mean body shape of forkbeard between the three geographical areas (Table 1). Fig 3 shows the differences between the mean shapes of forkbeard from the three areas and the overall mean shape. Specimens from Azores and mainland Portugal showed a relative displacement of anatomic landmarks 2, 3 and 7. A relative expansion of body height was observed in Madeira specimens. Both Madeira and Azores fish also showed a relative displacement of landmark 10 (posterior limit of the operculum).

**Table 1:** Procrustes distances between mean body shapes of the forkbeard from the three study areas from the Northeast Atlantic.

Area	Azores	Madeira
Madeira	0.0189 (0.0001)	-
Mainland Portugal	0.0209 (<0.0001)	0.0151 (0.0004)

Respective *p*-values, given in brackets, were obtained from permutation tests (10,000 permutation runs).

**Fig 4:**
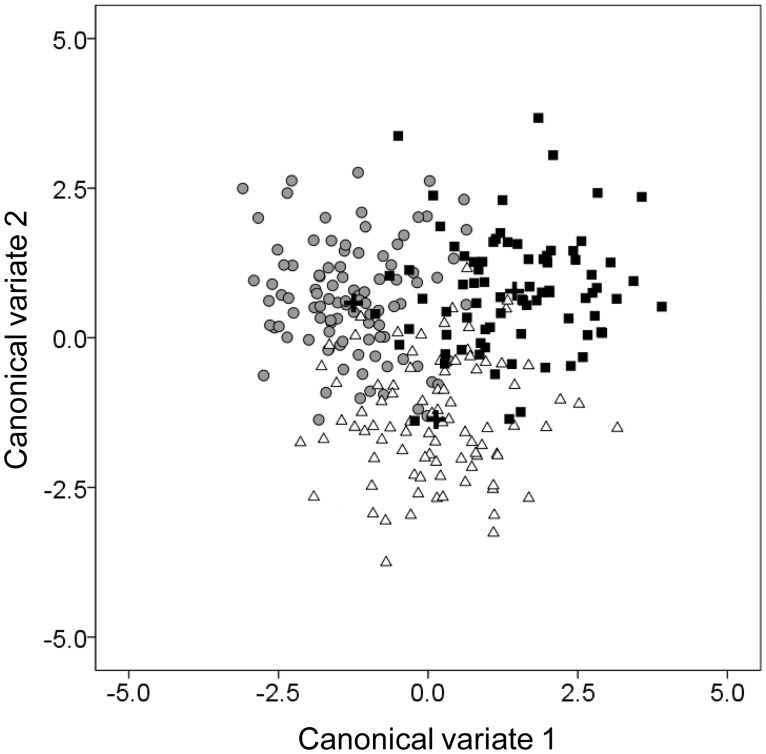
Two-dimensional ordination of forkbeard specimens from the three study areas from the Northeast Atlantic based on canonical variate analysis (CVA). Azores—black squares; Madeira—open triangles; mainland Portugal—grey circles. Plus signs indicate the class centroids.

The two first canonical variate functions were significant for the discrimination of specimens from the three study areas (*p* < 0.0001). Their score plot (Fig 4) shows a separation between the three areas, although some overlapping can be observed. The first two canonical variate functions explained 56.7% and 43.3% of between-group variance, respectively.

Assignment of forkbeard specimens to geographical areas correctly classified 77.6% of the total analysed specimens (λ = 0.234; κ = 0.663) (Table 2), meaning that a low misclassification occurred between the three study areas. The highest classification rate was found for mainland Portugal specimens with 81.2% classification success, while Azores showed the lowest classification success with 74.7%.

**Table 2:** Jackknife classification matrix of the discriminant canonical analysis applied to the forkbeard from Northeast Atlantic Ocean.

	n	Azores	Madeira	Mainland Portugal
Azores	79	**74.7%** **(59)**	13.9% (11)	11.4% (9)
Madeira	88	14.8% (13)	**76.1%** **(67)**	9.1% (8)
Mainland Portugal	101	5.9% (6)	12.9% (13)	**81.2%** **(82)**

Percentages in rows represent the classification into the areas given in columns (correct classification in bold). Number of specimens (n) correctly classified in brackets. Overall classification success: 77.6%, Wilk’s λ = 0.234, Cohen’s κ = 0.663.

### Genetic analysis

Within the 127 samples of forkbeard from the Northeast Atlantic and the Mediterranean, a total of 16 haplotypes of the mitochondrial cyt *b* gene fragment (GenBank accession numbers: KM252661-72 and KX215149-52) and only one haplotype of the nuclear S7 gene fragment (GenBank accession number KP005452) were found. The results of the latter gene were not shown due to its lack of genetic variation. Pseudogenes were not found in any of the samples sequenced, since two real peaks in the same position during the base calling in the chromatograms were never obtained.

The 846 bp amplified fragments of cyt *b* showed 17 polymorphic nucleotide positions and 8 of the 16 haplotypes were unique (Table 3). The Atlantic group showed 4 unique haplotypes (H3, H14 and H15 from Mainland Portugal, and H8 from Azores), all derived from the most commons haplotypes, and the Mediterranean group also exhibited 4 other unique haplotypes (H9, H10 and H16 from Croatia, and H12 from Spain), all being derived haplotypes. The most common haplotype (H1) was present in 67 specimens (~53% of total samples) and was found in all sampled geographic areas. The overall values of haplotype diversity (*h*) and nucleotide diversity (π) for the entire sample were 0.675 ± 0.038 and 0.00144 ± 0.00013, respectively. The Northeast Atlantic and the Mediterranean groups showed similar haplotype and nucleotide diversities (0.626 ± 0.054 and 0.00133 ± 0.00016; 0.749 ± 0.046 and 0.00165 ± 0.00022, respectively). In the Atlantic group, the highest haplotype richness was found in Mainland Portugal, where, for 27 samples, nine different haplotypes were present, and Azores showed the lowest one (5 different haplotypes were detected in 29 samples). In the Mediterranean Sea, the highest haplotype richness was found in Croatia, with 8 different haplotypes in 26 samples. A summary of the distribution of haplotypes per population and group, as well as a summary of haplotype and nucleotide diversities indices, are presented in Table 3.

**Table 3:** Summary of haplotypes distribution, and haplotype and nucleotide diversities for forkbeard populations.

Sampling sites	n	Number of polymorphic sites	Number of haplotypes	Haplotype diversity (*h*)	Nucleotide diversity (π)	Haplotypes															
						1	2	3	4	5	6	7	8	9	10	11	12	13	14	15	16
Mainland Portugal	27	9	9	0.687±0.095	0.00152±0.00030	15	3	1*	2	1	2							1	1*	1*	
Azores	29	6	5	0.677±0.070	0.00142±0.00025	15	5				6	2	1*								
Madeira	26	5	5	0.508±0.108	0.00107±0.00027	18	4				2					1		1			
*Northeast Atlantic Ocean*	*82*	*13*	*12*	*0*.*626±0*.*054*	*0*.*00133±0*.*00016*	*48*	*12*	1*	*2*	*1*	*10*	*2*	1*			*1*		*2*	1*	1*	
Croatia	26	8	8	0.772±0.064	0.00159±0.00028	11	3			1	6	2		1*	1*						1*
Spain	17	5	5	0.772±0.057	0.00189±0.00030	6	4				5					1	1*				
Italy	2	2	1	0.000±0.000	0.00000±0.00000	2															
*Mediterranean Sea*	*45*	*10*	*10*	*0*.*749±0*.*046*	*0*.*00165±0*.*00022*	*19*	*7*				*11*	*2*		1*	1*	*1*	1*				1*
**Total**	127	17	16	0.675±0.038	0.00144±0.00013	67	19	1	2	2	21	4	1	1	1	2	1	2	1	1	1

Sampling locations, with respective sample sizes (n), number of polymorphic sites, number of haplotypes and genetic diversity indices (*h* and π) haplotype distribution and unique haplotypes (*) per population and group for forkbeard populations from the Northeast Atlantic Ocean and the Mediterranean Sea.

**Fig 5:**
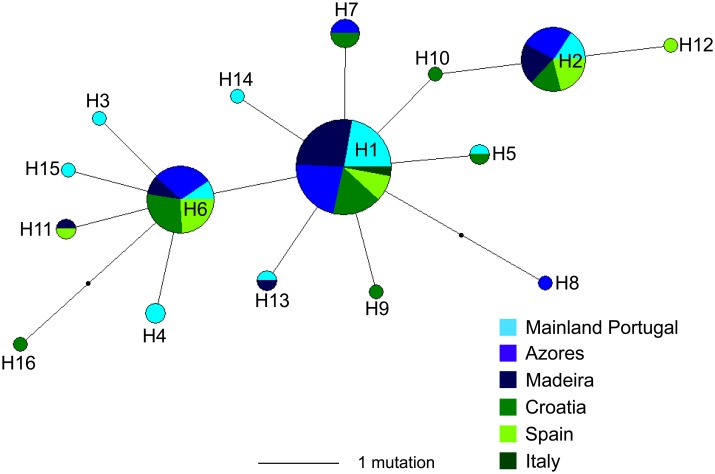
Median-joining haplotype network of the forkbeard from the Northeast Atlantic Ocean and the Mediterranean Sea for cytochrome *b* (846 bp). The size of each circle is proportional to the number of specimens carrying each haplotype, in which the smallest circles correspond to one individual, and the length of each line is proportional to the number of mutations.

The haplotype network did not show a phylogeographical structure among the sampled geographical region, presenting a “star-like” shape where the most common haplotype (H1) is shared between all sampled geographic areas, and the less frequent and unique haplotypes are connected to the central most common haplotype by only one or two mutation steps (Fig 5).

To assess the distribution of mtDNA variation, sampled geographic areas were pooled according to the two defined regional groups (Northeast Atlantic Ocean and Mediterranean Sea) and AMOVA was performed. AMOVA also revealed a lack of genetic structure, indicating that the overall source of variation was within populations (100.95%, *p* = 0.721) instead of among groups (0.43%, *p* = 0.301) (Table 4). The pairwise F-statistics (Table 5) showed no significant differentiation between the two regional groups.

**Table 4:** Analysis of molecular variance (AMOVA) among groups of the forkbeard based on mtDNA cyt *b*.

Source of variation	Total variance (%)	Fixation indices	*p*-value
Among groups	0.43	F_CT_ = 0.00139	0.301
Among populations within groups	-1.39	F_SC_ = -0.01395	0.763
Within populations	100.95	F_ST_ = -0.00954	0.721

AMOVA performed for two regional groups (Northeast Atlantic Ocean and Mediterranean Sea) of forkbeard based on mtDNA cyt *b* (10,000 permutations).

**Table 5:** Population pairwise FST values for cyt *b* sequences of the forkbeard.

F_ST_ values	Mainland Portugal	Azores	Madeira	Croatia	Spain	Italy
Mainland Portugal	-					
Azores	-0.01400	-				
Madeira	-0.01286	-0.01880	-			
Croatia	-0.01878	-0.02460	-0.00305	-		
Spain	0.02501	-0.00158	0.03581	0.00480	-	
Italy	-0.24769	-0.20916	-0.24432	-0.21304	-0.08410	-

FST values for cyt *b* below the diagonal.

*p*-values of all populations were not statistically significant (*p* > 0.05)

**Fig 6:**
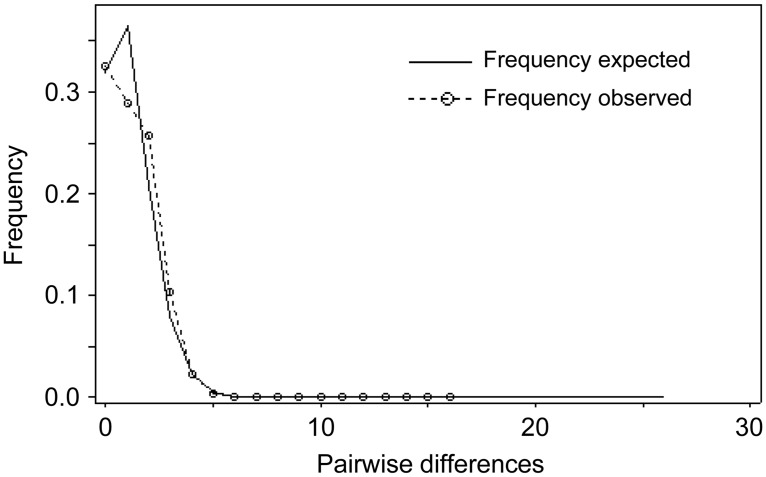
Frequency distributions of the number of pairwise nucleotide differences between cytochrome *b* haplotypes from forkbeard populations. Parameter values for the mismatch distributions are given in Table 6.

The distribution of pairwise nucleotide differences (mismatch distribution) showed a unimodal shape (Fig 6), suggesting that population range expansion may have occurred. The observed raggedness index was low and both P_SSD_ and P_RAG_ showed that the observed distributions did not differ significantly from those expected under a sudden expansion model (Table 6). Tajima’ D and Fu’s F tests were negative, although not statistically significant (Table 6), which do not support the population expansion hypothesis. The timing of demographic expansion for the forkbeard was estimated to have occurred at approximately 17,245 years ago (5,298–32,731 years ago).

**Table 6:** Parameters for the mismatch distribution and the neutrality tests of the forkbeard cyt *b* haplotypes.

Mismatch distribution analysis							Neutrality tests			
Parameters			Goodness-of-fit tests				Tajima’s *D* test		Fu’s *F* test	
θ_0_	θ_1_	τ	SSD	P_SSD_	Raggedness	P_RAG_	Tajima’s *D*	*p*-value	*F*	*p*-value
0.01582	16,953.38238	1.12630 (0.346–2.178)	0.06630	0.44050	0.06524	0.64267	-0.62883	0.39683	-1.46916	N.A.

Statistical significance of Tajima’s D and Fu’s F-statistics are presented. Numbers in parenthesis are the upper and lower bound of the 95% confidence interval (1,000 bootstrap replicates).

θ_0_ and θ_1_: pre-expansion and post-expansion population sizes; τ: time in number of generations elapsed since the sudden expansion episode; SSD: sum of squared deviations; Raggedness: raggedness index; P_SSD_ and P_RAG_: probability of expected mismatch distributions (1,000 bootstrap replicates) being significantly larger than observed mismatch distributions.

## Discussion

### Population structure and morphological differentiation

A substantial variation in forkbeard body shape was found, suggesting a clear separation between the three populations from the Northeast Atlantic. However, this morphological differentiation is not in accordance with the genetic population structure evidenced by the molecular marker used.

A large component of morphological variation in fishes is induced by exposure to different environmental factors during their development [68], namely food availability, temperature, salinity and prolonged swimming [33, 69]. Thus, the phenotypic plasticity of organisms may determine the phenotypic differentiation in populations of a species experiencing specific environmental conditions [33, 69]. As fish morphology is particularly dependent on environmental factors during early life stages, phenotypic differentiation may indicate that most fish from each population spent their lives in separate regions [33, 70] and provide an indirect assessment of population structure. However, it does not give direct evidence of genetic isolation between populations [35, 70]. Body shape and other morphological characters have long been used to delineate populations and continue to be used successfully [30–36], based on the fact that morphological features can relate to fitness and strong selective pressures that may underlie rapid genetic divergence between groups of fish [10].

Results of body geometric morphometric analysis obtained in this study suggests its usefulness as a population differentiation tool and indicate the existence of at least three phenotypically distinct local populations in the Northeast Atlantic, as reported by Vieira et al. [19] using otolith shape analysis. This population differentiation suggests a link between the levels of phenotypic divergence and geographical distance, and also a limited adult fish migration between populations from the Azores, Madeira and mainland Portugal. It is possible that different conditions (e.g. sea water temperature, depth, sediment type, food resources, predation risk, etc.) between the three study areas affect fish growth and behaviour [71], and thus, fish morphology. The discriminant analysis indicated that morphometric differentiation between the Azores and mainland Portugal was mainly due to differences in head characters of fishes, which may reflect differential habitat use [33] and may also be related to prey size [72, 73]. Madeira specimens showed a change in body depth that are mostly related to hydrodynamic and swimming performance [74–76], and locomotor adaptations to food capture and escape from predators [75, 76]. It is known that a higher body depth in benthic fish provides them a steeper power curve, furthering the prey capture and escape from predators (e.g. [74, 77, 78]).

Genetic data from cyt *b* suggest that the forkbeard forms a panmictic population along the Northeast Atlantic and the Mediterranean. *F*-statistics and AMOVA indicated no genetic structure over space, probably due to the high frequency of the most common haplotype (H1) (which minimizes the effect of rarer haplotypes), and also due to similarities between sequences caused by the low nucleotide diversity. These facts suggest that high levels of gene flow between geographically separated areas must have occurred in the past, or that the current distribution is an expanded area of an ancestral refugial and panmitic population, with low levels of diversity but widespread common/ancestral haplotypes. Moreover, and taking into account the existence of unique haplotypes in the Northeast Atlantic and the Mediterranean, recent gene flow between populations from these groups is unlikely to occur or, if present, occurs at low levels. Although larval behaviour of the forkbeard is unknown, other species of the genus *Phycis* present pelagic larvae for several months [79, 80] and, thus, long distance dispersal may occur during forkbeard larval phase. The putative absence or reduction of current larval dispersion of the forkbeard between the Northeast Atlantic and the Mediterranean suggests the presence of significant barriers to gene flow in this species. In fact, oceanographic and topographic features of the Atlantic, such as the patterns of circulation flow and water masses around seamounts, cause parcels of water to become trapped around these structures (e.g. Taylor columns) and retain larvae [81]. In addition, the large geographical distance between the European continental slope, the Azores (Mid-Atlantic Ridge) and Madeira may also create a barrier to dispersion of larvae [24] of this species.

### Demographic history

The genetic diversity pattern of the forkbeard might be consequence of population expansion after a period of low effective population size due to founder events or bottlenecks [82]. The phylogenetic relationships between mtDNA cyt *b* haplotypes was defined by a “star-like” network topology, in which rare haplotypes are derived from the most common haplotypes (H1, H2 and H6), presumably ancestors, frequently by one or two mutation steps. This could indicate that the forkbeard populations have recently expanded in size from one or from a small number of founders following a population bottleneck [59], suggesting a scenario of sudden population expansion in the Northeast Atlantic and the Mediterranean. Furthermore, mismatch distribution analysis also supports the hypothesis of population range expansion. The time of expansion estimated for the forkbeard was suggestive of a growing population since the end of the Last Glacial Maximum (LGM). Such demographic changes might have been caused by variations in the sea temperature and level during this time. Previous studies have suggested a strong historical influence on the genetic population structure of other gadiform fish species in the North Atlantic has (e.g. [83, 84]).

One of the major focuses in studies of phylogeography of the Northeast Atlantic specimens has been the location of refugia, where these organisms survived the successive glacial peaks during the Pleistocene [27]. These refugia most likely acted as propagation sources for the recolonization of more northerly areas during the interglacial periods [85]. Most studies consider the Pleistocene and in particular the LGM to have been critical times when populations were not present in the frozen north and went through severe bottlenecks in their refuge habitats [86]. The forkbeard has a subtropical geographical distribution, exhibiting a reduced temperature tolerance (12–15°C, ~300 m depth). During the last glaciation and due to the progression of the polar front, this species most likely has been pushed to southern limits of its distribution to seek refugia with similar temperature to its optimum. Based on this, and on the haplotype diversity presented by the forkbeard populations and the generated network, two possible scenarios of population expansion could be delineated. In the first scenario, during the LGM, forkbeard populations took refuge in the Mediterranean Sea and, after the end of this period, the species expanded to the northern and southern Northeast Atlantic. However, and according to this scenario, the Mediterranean group should show much higher haplotype diversity than Atlantic one. On the other hand, the Mediterranean LGM sea temperatures decreased drastically, being low enough to support populations of northern gadids and Atlantic salmon [85], but never to support forkbeard populations. Thus, this scenario is unlikely to have occurred. In the second and most likely expansion scenario, during the LGM the southern extreme of forkbeard distribution (Cape Verde region) served as refugium for haplotypes H1, H2 and H6, and, after the glaciation, the species expanded northward colonizing the rest of the Northeast Atlantic and the Mediterranean. The presence of the most common haplotype (H1) in all sampled geographic areas corroborates this hypothesis. Then, and although the gene flow between the Atlantic and the Mediterranean groups may have occurred, each group began its own process of divergence as suggested by the presence of four unique haplotypes in both the Atlantic and the Mediterranean groups. In fact, the oceanographic and topographic features of the Atlantic [81], and the large geographical distance between the Azores, Madeira and the European continental slope [24], seems to have limited the gene flow between the Northeast Atlantic and the Mediterranean populations of the forkbeard, leading to the emergence of unique haplotypes in each region. This hypothesis of the southern extreme refugium can also be reinforced by the similarity of sea temperature in the Western Sahara coast, Cape Verde and Mauritanian coast during the last glaciation [87, 88], and the current forkbeard’s thermal belt. With the rising of sea level in interglacial periods, new areas could be colonized, enabling the forkbeard to extend its ranges and resulting in spatial and demographic and expansions. In agreement, the Atlantic tropical cost of Africa has previously been proposed as possible refugium during the glacial periods for fish species of the Northeast Atlantic (e.g. bleniids [26, 89], *Chromis limbata* [90]).

### Final considerations and implications for fishery management

At the present, little information exists about the population structure of the forkbeard and from a management point of view the knowledge of this issue is crucial.

Genetic data suggest that the forkbeard forms a panmictic population in the Northeast Atlantic and the Mediterranean, and body shape variations indicate, as otolith shape analysis had already shown [19], a clear separation between the three populations from the Northeast Atlantic. The absence of a clear genetic structure and the differences in forkbeard morphology are not contradictory, since neutral genetic markers were used in this study. In fact, similar results have already been obtained by other authors studying different animal groups like molluscs (e.g. [2]) and fish (e.g. [34, 91, 92]). However, the existence of unique haplotypes in the Northeast Atlantic and the Mediterranean suggests that recent gene flow between populations from these groups should be limited. These facts suggest that gene flow between geographically separated areas must have occurred in the past, probably during the larval phase of the species, being more restricted in the present. Furthermore, a prolonged separation of post-larval fish in different environmental regimes leads to phenotypic plasticity in response to distinct selective pressures or environmental constraints.

The information presented in this study shows that, along its distribution area, the forkbeard presents, at least, three phenotypic stocks—mainland Portugal, Azores and Madeira, in the southern NE Atlantic, and two genotypic stocks between the NE Atlantic and the Mediterranean. Any depletion in one of these populations might not be compensated by migration from the others, at least at a sufficiently rapid rate to ensure resource sustainability. The most precautionary management approach should be to consider the different populations of the forkbeard from the Northeast Atlantic and the Mediterranean as separate stocks, to ensure sustainability of resources and genetic biodiversity maintenance [13]. A failure to employ the knowledge on the population structure in the management of complex fish stocks, such as in the case of the forkbeard, may originate incorrect management actions that may lead to overexploitation of some stocks.

Future studies using a holistic approach, which employ a broad spectrum of complementary techniques (e.g. otolith chemical composition and parasites as biological tags) [13], as well as the use of more sensitive markers such as microsatellites, or adaptive markers like candidate genes (e.g. quantitative trait loci associated with body shape, such as genes coding the a-3 subunit of Collagen VI [93] and a glycerol kinase [94]), will be necessary to complementary address this issue.
